# Convention of Teachers of Medical Colleges in the United States

**Published:** 1867-06

**Authors:** 


### CONVENTION OF TEACHERS OF MEDICAL COLLEGES IN THE UNITED STATES.

In pursuance of a call of a Committee appointed by the American Medical Association, at its last session, held in Baltimore, May 3d, 1866, delegates from most of the medical institutions of the country met Friday morning, May 3d, at 10 o'clock in the forenoon, in the faculty-room of the Medical College of Ohio.

Professor Davis, of Chicago, Chairman of said Committee, called the meeting to order, and, after stating its object, proposed with a view to facilitate a permanent organization, the appointment of a temporary Chairman and Secretary. This proposition being accepted, Prof. A. Stillé, of Philadelphia, was elected temporary Chairman, and Prof. G. C. E. Weber, of Cleveland, Ohio, Secretary.

Prof. Davis, of Chicago, Prof. Donaldson, of Baltimore, and Prof. Blackman, of Cincinnati, were appointed a Committee on Credentials. The following-named gentlemen were approved as delegates from their respective colleges:—

A Stillé, from the University of Pennsylvania; S. D. Gross, from Jefferson Medical College, Philadelphia; F. Donaldson, from the University of Maryland, Baltimore; F. Howard, from Georgetown Medical College, Washington City; James M. Holloway, from University of Louisville, Ky.; A. Hammer and A. J. Steele, from Humboldt Medical College, St. Louis; J. N. McDowell, from Missouri Medical College, St. Louis; J. C. Hughes and M. K. Taylor, from the Medical Department of Iowa University, Keokuk; W. H. Byford and N. S. Davis, from Chicago Medical College, Chicago; A. B. Palmer, from the Medical Department of University of Michigan, Ann Arbor; Alden March, from Albany Medical College, N.Y.; A. B. Palmer, from Berkshire Medical College, Pittsfield, Mass.; G. C. E. Weber, from Charity Hospital Medical College, Cleveland; Francis Carter, from Starling Medical College, Columbus; Geo. C. Blackman and G. C. Comegys, from Medical College of Ohio; Geo. Mendenhall and E. B. Stevens, from Miami Medical College, Cincinnati; B. L. Lawson and — Read, from Cincinnati College of Medicine; and T. M. Logan, from Toland Medical College, San Francisco, Cal.

Prof. Stillé, of Philadelphia, was chosen permanent Chairman, and Prof. Weber, of Cleveland, Secretary.

Profs. Holloway, of Louisville; Davis, of Chicago; Blackman, of Cincinnati; and March, of Albany, were appointed a committee to report on the order of the different subjects which were to occupy the attention of the Convention.

After which the Convention adjourned to 4 o'clock P.M.

FIRST DAY—*Afternoon Session.*

The Convention was called to order at 4 o'clock P.M.

Dr. N. S. Davis, from the committee to present a proper order of business, reported the following distinct propositions for the consideration of the Convention:—

"1. That every student applying for matriculation in a Medical College, shall be required to show, either by satisfactory certificates, or by a direct examination by a committee of the faculty, that he possesses a thorough knowledge of the common English branches of education, including the first series of mathematics and the natural sciences, and the certificates presented, or the results of the examinations thus required, be regularly filed, as a part of the records of each Medical College.

"2. That every medical student be required to study not only three full years, but also to attend three regular annual courses of medical college instruction before being admitted to an examination for the degree of Doctor of Medicine.

"3. That the *minimum* duration of a regular annual lecture term, or course of medical college instruction, shall be five calendar months.

"4. That every medical college shall embrace in its curriculum at least *thirteen* professorships, including substantially the following branches, namely: Descriptive Anatomy, Physiology and Histology, Inorganic Chemistry, Materia Medica, Organic Chemistry and Toxicology, General Pathology and Public Hygiene, Surgical Anatomy and Operations of Surgery, Medical Jurisprudence, Practice of Medicine, Practice of Surgery, Obstetrics and Diseases of Women, Clinical Medicine and Clinical Surgery. That these several branches shall be divided into three groups or series, corresponding with the three years required for medical study. The first, or freshman series, shall embrace Descriptive Anatomy, Physiology and Histology, Inorganic Chemistry, and Materia Medica. To these, the attention of the student shall be mainly restricted during the first year of his studies, and on them he shall be thoroughly examined by the proper members of the faculty at the close of his first course of medical college instruction, and receive a certificate indicating the degree of his progress. The second, or junior series, shall embrace Organic Chemistry and Toxicology, General Pathology, Public Hygiene, Surgical Anatomy and Operations of Surgery, and Medical Jurisprudence. To these, the attention of the medical student shall be directed during the second year of his studies, and on them he shall be examined at the close of his second course of medical college instruction, the same as after the first. The third, or senior series, shall embrace Practical Medicine, Practical Surgery, Obstetrics and Diseases of Women, with Clinical Medicine and Clinical Surgery in a hospital. These shall occupy the attention of the student during the third year of his medical studies, and at the close of the third course of medical college attendance, he shall undergo a general examination in all the departments, as a prerequisite for the degree of Doctor of Medicine.

"The instruction in the three series of branches is to be given simultaneously, and to continue throughout the whole of each annual college term; each student attending the lectures on such branches as belong to his period of progress in study, in the same manner as the Sophomore, Junior, and Senior classes each pursue their respective studies simultaneously throughout the collegiate year, in all our literary colleges.

"5. That the practice of selling individual tickets by members of medical college faculties should be abolished, and, in place of it, each stndent should be charged a specified sum for each annual course of medical college instruction; the sum being the same for each of the three courses before graduating; and any student or practitioner who has attended three full courses in any one college, shall be entitled to attend any subsequent course or courses in that college gratuitously. The fees paid for each annual course of college instruction should be paid to the Treasurer of the college, and subsequently distributed to each member of the faculty, at such time and in such proportion as the Trustees and Faculty of each college shall determine.

"6. That inasmuch as the maintenance of an efficient medical college requires a large expenditure of money annually, and inasmuch as there is no reasonable hope of adequate endowments from the several State governments, the exaction of a just and reasonable annual lecture fee is a necessity with which all medical colleges should comply, and that $105 should be the minimum fee for each regular annual course of instruction in any medical college in the United States."

The first proposition was taken up and discussed by Professors Davis, Gross, Comegys, McDowell, Hammer, Taylor, and Palmer, and with an amendment, so as to strike out the words "natural sciences," and add "sufficient knowledge of Latin and Greek to understand the technical terms of the profession," it was adopted.

The Convention then adjourned, to meet at 9½ o'clock Saturday morning.

SECOND DAY—*Morning Session.*

On Saturday, 4th inst., the Convention was called to order by the Chairman, Professor Stillé. The minutes of the preceding session were read and adopted. The Chair then announced that the next business in order was the discussion of Section 2 of the Report of the Committee on the order of Business, which reads as follows:—

"That every medical student be required to study three full years, including three regular annual courses of medical college instruction, before being admitted to an examination for the degree of Doctor of Medicine."

Professor Gross, of Philadelphia, moved to amend, so as to insert "four" after study, instead of "three."

Remarks were made by Professors Gross; Hammer, of St.

Louis; Davis, of Chicago; Palmer, of Michigan; and McDowell of St. Louis.

The Convention then suspended the rules, for the purpose of allowing Professor Davis to introduce the following resolution: "*Resolved*, That on all distinct propositions under the consideration of this Convention, no member shall speak more than once, until all other members have spoken who wish to speak." Adopted.

Prof. F. Howard, of Washington City, moved to amend, by inserting "not less than three years," instead of "three full years." Lost.

Prof. Gross' amendment was then adopted.

On motion of Prof. Gross, the entire section, as amended, was unanimously adopted.

Prof. Hammar moved to take up for consideration Section 4, prior to Section 3. Lost.

Section 3 was read, viz., "That the minimum duration of a regular annual lecture term or course of medical college instruction shall be five calendar months."

Prof. Gross moved to amend by inserting "six" in place of "five calendar months." Carried.

Section 3, as amended, was then adopted.

Section 4, being next in order, came up for discussion. Prof. Gross moved to discuss the different parts of this section separately. First, that relating to the different branches recommended to be taught in the schools. Second, the number of professorships. Third, the division of studies. Adopted.

Prof. Hammar moved to add to the different branches, Natural Philosophy and Pathological Anatomy.

Prof. Donaldson, of Baltimore, moved to act upon these propositions separately.

The vote on the addition of Natural Philosophy being taken, it was rejected.

The amendment adding Pathological Anatomy was carried.

Prof. Byford, of Chicago, moved to amend by including Diseases of Children. Carried.

On motion, the Convention then adjourned to meet at 4 o'clock, P.M.

SECOND DAY—*Afternoon Session.*.

The meeting having been called to order by the chairman, the second part of Section 4 was called up for discussion.

Prof. Gross moved to amend by inserting after the words "following branches," "to be taught by not less than nine professors." Carried.

Remarks were made by Professors Gross, Palmer, Davis, Hammar, Howard, and Taylor.

The third part of Section 4, referring to the division of studies, was next considered.

Prof. Davis moved to amend, by making that part read as follows:—

"That these several branches shall be divided into three groups or series, corresponding with the three courses of medical college instruction required.

The first, or Freshman series, shall embrace Descriptive Anatomy and Practical Dissections, Physiology and Histology, Inorganic Chemistry, Materia Medica and Therapeutics.

"To these, the attention of the student shall be mainly restricted during his first course of medical college instruction, and in these he shall submit to a thorough examination by the proper members of the Faculty at its close, and receive a certificate indicating the degree of his progress.

"The second, or Junior series, shall embrace organic Chemistry and Toxicology, General Pathology, Pathological Anatomy and Public Hygiene, Surgical Anatomy and Operations of Surgery, and Medical Jurisprudence. To these, the attention of the medical student shall be directed during his second course of medical college instruction, and in them he shall be examined at the close of his second course, in the same manner as after the first.

"The third, or Senior series, shall embrace Practical Medicine, Practical Surgery, Obstetrics, and Diseases peculiar to Women and Children; with Clinical Medicine and Clinical Surgery in a hospital. These shall occupy the attention of the student during his third course of medical college instruction, and at its close he shall be eligible to a general examination on all the branches, as a prerequisite for the degree of Doctor of Medicine. The instruction in the three series of branches is to be given simultaneously, and to continue throughout the whole of each annual college term; each student attending the lectures on such branches as belong to his period of progress in study, in the same manner as the Sophomore, Junior, and Senior classes each pursue their respective studies simultaneously throughout the collegiate year, in all our literary colleges."

After a prolonged debate, in which Professors Gross, Palmer, Blackman, Davis, and Taylor participated, the motion of Prof. Davis prevailed.

Prof. Davis then moved the adoption of the entire section as amended. Carried.

Section 5 was then taken up, and, upon motion of Professor Palmer, was laid on the table.

Section 6, being in order, was read, but, on motion of Prof. Gross, was also laid upon the table until Monday morning, 6th inst.

On motion of Prof. Davis, the Convention then adjourned to meet at 10 A.M., on Monday morning.

THIRD DAY—*Morning Session.*

The meeting was called to order by Prof. Stillé, at 10 o'clock A.M. The minutes of the previous session were read and approved.

The Committee on Credentials announced Dr. T. M. Logan, of Sacramento, California, as an authorized delegate from the Faculty of the Toland Medical College of San Francisco.

Prof. Gross moved to reconsider parts of Section 4, relating to the branches to be taught in medical colleges.

Prof. Hammar moved to suspend the rules for that purpose. Carried.

Prof. Gross moved to amend part 1st, Section 4, by inserting the words "Medical Ethics" after the words "Medical Jurisprudence.

Prof. Palmer moved the adoption of the amendment. Carried.

Prof. Comegys moved the reconsideration of Section 1.

After suspension of the rules, this motion was adopted.

Prof. Comegys moved to amend Section 1, by inserting "Elements of Natural Sciences" after the word "Mathematics." Carried.

Prof. Hammar moved the adoption of the whole section as amended. Carried.

Section 6 was then considered.

On motion of Prof. Donaldson, it was laid on the table.

Prof. Palmer then introduced the following resolution:—

*Resolved*, That every medical college should immediately adopt some effectual method of ascertaining the actual attendance of students upon its lectures and other exercises, and at the close of each session or of the attendance of the student a certificate, specifying the time and the courses of instruction actually attended, should be given; and such certificate only should be received by other colleges as evidence of such attendance."

The resolution was adopted.

Prof. Davis moved the adoption of all the sections as amended.

Prof. Gross moved to transmit a copy of these sections, as adopted by this Convention, certified to by its officers, to the American Medical Association, at its next session.

Prof. Davis then introduced the following resolution:—

"*Resolved*, That a committee of five be appointed by the President, whose duty it shall be to present the several propositions adopted by the Convention to the trustees and faculties of all the medical colleges in this country, and solicit their definite action thereon, with a view to the early and simultaneous practical adoption of the same throughout the whole country, And that the same committee be authorized to call another convention, whenever deemed advisable."

The Chair appointed the following gentlemen that committee: Profs. Davis, of Chicago; Donaldson, of Baltimore; Gross, of Philadelphia; March, of Albany; Blackman, of Cincinnati.

The Chairman then introduced Dr. Vattier, President of the Cincinnati Academy of Medicine, who invited the members of the Convention to be present at the opening of the Academy in the evening.

Prof. March moved to accept the invitation. Carried.

On motion of Prof. Davis, a vote of thanks was returned to the Chairman and Secretary of the Convention, for the efficiency with which they have discharged their duties, and to the Faculty of the Ohio Medical College for the use of their hall.

The President returned his thanks to the members of the Convention, in a neat and appropriate speech.

Prof. Stevens moved that a formal written thesis on some professional topic shall still be regarded as one of the indispensible requirements of the doctorate.

Remarks were made by Professors Comegys, Stevens, and Donaldson.

Prof. Davis then rose, simply to suggest whether there was not some danger of entering upon the consideration of propositions involving details that might unnecessarily complicate the great leading object for which we have been laboring. Whether the time-honored and universal custom of requiring the medical students to write a thesis should be insisted on or not, would have but little bearing on the great principles involved in the revision of our system of medical education. If the standard of preliminary education which we have here adopted should be carried into effect, it would remove one of the objects for which the writing of a thesis was originally demanded. Yet, he said, it was desirable to retain the practice, if for no other purpose than to encourage every student in the habit of expressing his thoughts on paper. But the great and all-important object of this Convention was simply to place the system of medical education in this country upon sound educational principles, by erecting a standard of preliminary education, by increasing the period of study, by adding to the college courses, and by determining a rational order of study. This we have now done, so far as this Convention is concerned, by the harmonious adoption of the five propositions already passed upon. And he earnestly suggested whether we had not better stop here, and devote the remainder of our time to the work of devising the most efficient means to secure the adoption and simultaneous practical execution of the provisions already agreed upon by all the colleges of our country, and leave all minor matters of detail to be determined as time and circumstances should indicate in the future.

Thereupon, on motion of Prof. Hammar, the Convention adjourned, subject to the call of the Committee.

The following is a correct copy of the several propositions as finally adopted by the *unanimous* vote of the Convention;—

"*Resolved:*—

1st. That every student applying for matriculation in a Medical College, shall be required to show, either by satisfactory certificate, or by a direct examination by a Committee of the Faculty, that he possesses a thorough knowledge of the common English branches of education, including the first series of mathematics, the elements of natural sciences, and a sufficient knowledge of Latin and Greek to understand the technical terms of the profession; and that the certificate presented, or the result of the examinations thus required, be regularly filed as a part of the records of each Medical College.

2d. That every Medical Student shall be required to study four full years, including three regular annual courses of Medical College instruction, before being admitted to an examination for the degree of Doctor of Medicine.

3d. That the minimum duration of a regular annual lecture term, or course of Medical College instruction, shall be six calendar months.

4th. That every Medical College shall embrace in its curriculum the following branches, to be taught by not less than nine Professors, namely:—Descriptive anatomy, including dissections; physiology and histology; inorganic chemistry, materia medica, organic chemistry, and toxicology; general pathology, therapeutics, pathological anatomy, and public hygiene; surgical anatomy and operations of surgery; medical jurisprudence and medical ethics; practice of medicine, practice of surgery, obstetrics, and diseases of women and children; clinical medicine and clinical surgery. And that these several branches shall be divided into three groups or series, corresponding with the three courses of Medical College instruction required.

The first, or *Freshman Series*, shall embrace descriptive anatomy and practical bisections; physiology and histology; inorganic chemistry, and materia medica. To these, the attention of the student shall be mainly restricted during his first course of Medical College instruction, and in these he shall submit to a thorough examination, by the proper members of the Faculty, at its close, and receive a certificate indicating the degree of his progress.

The second, or *Junior Series*, shall embrace organic chemistry and toxicology; general pathology, pathological anatomy, therapeutics, and public hygiene; surgical anatomy and operations of surgery; medical jurisprudence and medical ethics. To these, the attention of the medical student shall be directed during his second course of Medical College instruction, and in them he shall be examined, at the close of his second course, in the same manner as after the first.

The third, or *Senior Series*, shall embrace practical medicine; practical surgery; obstetrics, and diseases peculiar to women and children; with clinical medicine and clinical surgery in a hospital. These shall occupy the attention of the student during his third course of College instruction, and at its close he shall be eligible to a general examination for the degree of Doctor of Medicine.

The instruction in the three series is to be given simultaneously, and to continue throughout the whole of each annual College term; each student attending the lectures on such branches as belong to his period of progress in study, in the same manner as the sophomore, junior, and senior classes, each pursue their studies simultaneously throughout the collegiate year, in all our Literary Colleges.

5th. That every Medical College should immediately adopt same effectual method of ascertaining the actual attendance of students, upon its lectures and other exercises, and at the close of each session, of the attendance of the student, a certificate, specifying the time and the courses of instruction actually attended, should be given, and such certificate only should be received by other Colleges as evidence of such attendance.

6th. That a committee of five be appointed by the President, whose duty it shall be to present the several propositions adopted by this Convention, to the Trustees and Faculties of all he Medical Colleges in this country, and solicit their definite ction thereon, with a view to the early and simultaneous pracical adoption of the same throughout the whole country. And hat the same committee be authorized to call another conveni on whenever deemed advisable."

